# Cryotherapy Treatment Effect on Heat-Treated Nickel-Titanium (NiTi) Rotary Single-File System: An In Vitro Comparative Study by Finite Element Analysis

**DOI:** 10.7759/cureus.60271

**Published:** 2024-05-14

**Authors:** Yadav Chakaravarthy, Vishnu Prasad, Vanita Dattatray Revankar, Haripriya Karthikeyan, Suresh Saravanan, Aishwarya Santosh

**Affiliations:** 1 Conservative Dentistry and Endodontics, Vinayaka Mission's Sankarachariyar Dental College, Salem, IND; 2 Conservative Dentistry and Endodontics, Chettinad Dental College and Research Institute, Kelambakkam, IND; 3 Conservative Dentistry and Endodontics, Sri Venkateshwaraa Dental College, Pondicherry, IND; 4 Conservative Dentistry and Endodontics, Kavita Dental Clinic, Bangalore, IND

**Keywords:** komet, procodile, nickle-titanium single rotary file, finite elemental analysis, cryotherapy

## Abstract

Background

The use of endodontic files multiple times can cause fatigue in them and can lead to their separation in the root canal. The purpose of this study was to achieve a reduction in cyclic fatigue stress in a newly introduced nickel-titanium (NiTi) rotary single-file system. The study aimed to determine whether cryotherapy could help reduce cyclic fatigue and stress on rotary files after multiple uses during root canal treatment. By utilizing finite element analysis (FEA), the study provided a comprehensive evaluation of how cryotherapy might enhance the performance and longevity of these instruments, ultimately benefiting patients undergoing root canal therapy.

Methodology

This in vitro comparative study used scanned plastic teeth with genuine root canal anatomy and FEA to investigate the mechanical response to cyclic fatigue and stress of NiTi rotary file system. The endodontic file (Procodile, Komet) was created through the complex root canal geometries, for which mandibular tooth models were scanned and created by a computer software (IDEAS11 NX; UGSiPlano, TX). The total sample size was 34, divided into two groups, with each group comprising 17 participants (n = 17). The results were analyzed by analysis of variance (ANOVA) test.

Results

The results revealed that the p-values were more than 0.525, indicating no significant reduction in cyclic fatigue when the NiTi rotary single-file system (Procodile, Komet) was treated with cryotherapy (eight cycles). However, stress reduction was observed in the NiTi rotary single-file system when it was treated with cryotherapy.

Conclusion

This in vitro comparative study concluded that cryotherapy helps to reduce the stress of NiTi rotary single-file system. Nonetheless, more research is needed to understand the clinical significance of the findings of the current in vitro study.

## Introduction

By removing inflammatory or necrotic pulp tissue from the root canal system, endodontic treatment known as root canal therapy aims to eradicate infection and maintain the original tooth. Typically, this process entails shaping and cleaning the root canal using specialized endodontic rotary devices before filling it with biocompatible material. The orientation and position of possible root fractures during treatment can be affected by several essential variables, such as dentin thickness, external root morphology, and root canal shape [1].

Scientific research does not provide substantial support for the generally held idea that teeth with root canal therapy are more prone to fractures than healthy teeth. According to recent research, the fracture rate of teeth with root canal therapy and teeth without endodontic treatment is similar, indicating that the process may not have a significant negative impact on the mechanical characteristics of teeth [2,3].

In recent years, the design and composition of endodontic rotary files have seen significant advancements, with changes in the alloys' chemical properties and the files' geometric design. These developments have improved the mechanical performance and durability of these instruments [4]. Despite recent developments, the unexpected separation of endodontic rotary files within the root canal during treatment remains difficult for endodontists [5,6].

To better understand and address these challenges, endodontics has leveraged the power of finite element analysis (FEA) to assess stress distribution and potential points of failure during root canal procedures. This type of analysis provides valuable insights into how different factors affect the performance of rotary files and the overall success of the treatment [7-9].

In an effort to further improve endodontic outcomes, an in vitro comparative study was conducted to examine the potential benefits of applying cryotherapy to a rotary single-file system (Procodile, Komet) [10-12]. The study aimed to determine whether cryotherapy could help reduce cyclic fatigue and stress on rotary files after multiple uses during root canal treatment. By utilizing FEA, the study provided a comprehensive evaluation of how cryotherapy might enhance the performance and longevity of these instruments, ultimately benefiting patients undergoing root canal therapy.

## Materials and methods

Study setting and design

This in vitro comparative study utilized scanned plastic teeth with genuine root canal anatomy and FEA to investigate the nickel-titanium (NiTi) rotary file system's mechanical response to cyclic fatigue stress. The scanned plastic tooth was shaped to apical size #20 by the newly introduced NiTi rotary single-file system (Procodile, Komet) instrument. The endodontic file (Procodile, Komet) was created by computer software (FEM software) through the junction of two distinct geometries. Mandibular tooth models were scanned and developed by FEM (IDEAS11 NX; UGSiPlano, TX). Consent was obtained or waived by all participants in this study. Institutional Ethics Committee for Research on Human Subjects of Vinayaka Mission's Sankarachariyar Dental College issued approval RES/VMSDC/2022/02.

Data sources and variables 

In this invitro study, the experiment group was divided into Group A and Group B. Group A included the FEA of the NiTi rotary single-file system (Procodile, Komet), which was non-cryotreated. It was further subdivided into three subgroups: subgroup (A1), which included cryotherapy untreated NITI rotary single-file system (Procodile, Komet) that evaluated cyclic fatigue after two times of biomechanical preparation; subgroup (A2), which had cryotherapy untreated NiTi rotary single-file system (Procodile, Komet) that evaluated cyclic fatigue after four times of biomechanical preparation; and subgroup (A3), incorporated cryotherapy untreated NITI rotary single-file system( Procodile, Komet) that evaluated cyclic fatigue after six times of biomechanical preparation. Group B displayed FEA of NiTi rotary single-file system (Procodile, Komet) which was cryotreated. It was further divided into three subgroups: subgroup (B1), which comprised of cryotherapy treated NITI rotary single-file system (Procodile, Komet) that evaluated cyclic fatigue after two times of biomechanical preparation; subgroup (B2), which constituted cryotherapy treated NITI rotary single-file system (Procodile, Komet) that evaluated cyclic fatigue after four times of biomechanical preparation; and subgroup (B3), comprised of cryotherapy treated NiTi rotary single-file system (Procodile, Komet) that evaluated cyclic fatigue after six times of biomechanical preparation. This endodontic file has three distinct components, namely, active, shaft, and transition. The computer generated this endodontic file by joining two different geometries: the machined and raw materials. There is a direct overload on this reference node. Two different load cases were created in order to investigate bending and torsional stresses. The FE solver receives an input file containing the whole FE model definition through this procedure. It is important to emphasize that this approach eliminates the need for an extra third-party software by producing fully parameterized FE models in which the user may specify the geometry mesh density, material properties, and loading conditions. To generate a 3D model for FE analysis, a mesh of linear, eight-noded, hexahedral components was put over the instrument in the program. One economical alternative is cryogenic therapy.

Instead of only changing the metal's surface, cryotreatment has the ability to change the metal's whole cross-section. The methodology suggested by Vinothkumar was adhered to in this in vitro investigation [13]. The cryogenic liquid was turned into vapor and entered the area below the platform in accordance with a time-temperature program that cooled the samples with evaporating vapor from the cryogenic liquid pool in the area below the platform, bringing the temperature down gradually to -184.44°C (88.6 K or -300°F) at a rate of 1.5°C/min for two hours and 18 minutes. After cryogenically treating NiTi instruments, their microhardness was improved.

**Figure 1:**
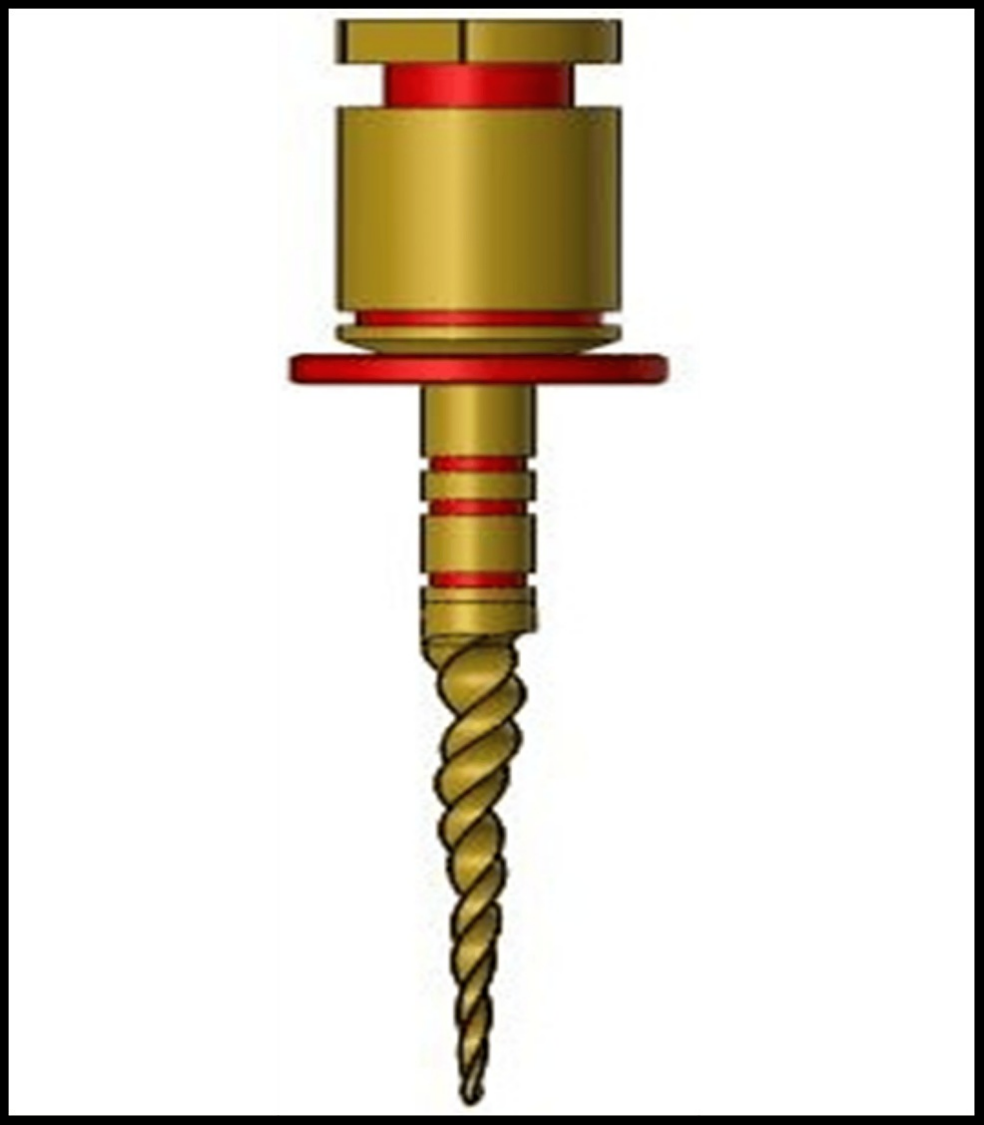
Procodile image

At a higher temperature, it was discovered that the cryogenic treatment had more positive effects than the conventional cold treatment, such as improved metal overall strength and cutting efficiency. Numerous details on the stress conditions along the file length are provided by the maximum and average stress profiles. This indicates maximum localized stress levels and the average stress in each section of NiTi rotary file system. An image of Procodile file has been shown in Figure 1.

Data analysis

The data was initially collected in the Kobo Toolkit. It was then exported to MS Excel (Microsoft Corporation, Redmond, Washington, United States) for analysis. The mean and standard deviation of the variables were calculated and tabulated. Student's t-test was used, and a corresponding p-value was derived. A p-value less than 0.05 was considered significant. 

## Results

Table 1 shows that the comparison between the Procodile file and the Procodile-cryotreated file under fatigue conditions reveals a significant difference in performance. The Procodile file endured 4.022 cycles, while the Procodile-cryotreated file demonstrated almost double the endurance, completing 8.218 cycles. This data suggests that cryotreatment may enhance the durability of the Procodile file, making it more resistant to fatigue.

**Table 1:** Comparison of the number of cycles between Procodile and Procodile-cryotreated files

Load cases	No. of cycles	
	Procodile file	Procodile-cryotreated file
Fatigue	4.022	8.218

The comparison of file thread displacement between Procodile and Procodile-cryotreated files (Table 2) reveals the statistical significance of the differences. Both groups had 17 samples. The mean thread displacement for the Procodile file was 3.06 mm, with a standard deviation (SD) of 3.07 and a standard error (SE) of 0.75. For the Procodile-cryotreated file, the mean thread displacement was 3.83 mm, with an SD of 3.84 and an SE of 0.93. The t-value was 0.642, resulting in a p-value of 0.525. Therefore, the data suggests no significant difference in the file thread displacement between the two groups. 

**Table 2:** p-value between Procodile and Procodile-cryotreated files p-value < 0.05 is considered to be significant. N is the number of patients

File thread displacement (mm)	N	Mean	Standard deviation	SE	t-value	p-value	Remarks
Procodile file	17	3.06	3.07	0.75	0.642	0.525	Not significant
Procodile-cryotreated file	17	3.83	3.84	0.93			

Table 3 compares stress levels between the Procodile file and the Procodile-cryotreated file during a bending test. The stress experienced by the Procodile file was 1186.04 MPa, whereas the Procodile-cryotreated file endured a lower stress level of 948.84 MPa. This stress reduction suggests that the cryotreated file may offer improved resilience and better performance under bending stress than the standard Procodile file.

**Table 3:** Comparison of stress between Procodile and Procodile -cryotreated files Mpa: MegaPascal

Load cases	Stress (Mpa)	
	Procodile file	Procodile-cryotreated file
Bending test	1186.04	948.84

## Discussion

The recent introduction of a single-file rotary system has claimed to reduce the stress on files and prevent separation. Compared to many file systems, a single-file system improves the root canal's shaping ability and cyclic fatigue resistance [14,15]. NiTi has the characteristics of two distinct metals and may exist in two crystalline forms. The alloy typically exists under steady-state stress, transforming into a martensite structure in an austenitic crystalline phase. Nevertheless, the structure reverts to its original austenitic phase and form when the stress is released. Stress-induced thermoelastic transition is used to describe this phenomenon [11].

Over the years, numerous methods have been tried to analyze stress on the NiTi rotary files. FEA is a computer-based study system that helps determine the stress distribution in rotary files. It is based on the principle that the structure of a rotary file is divided into a small number of elements where the mechanical behavior is connected by carrier points called “nodes.” Cryogenic treatment is a cost-effective option. The principle of cryotherapy involved loading the alloy's austenitic phase and changing it to the alloy's martensitic phase. Due to changes in the crystalline nature of the metal in the rotary file caused by cryotherapy help, the martensitic phase of the alloy changes to the austenitic phase, thereby helping to reduce the stress of a rotary file [13]. Despite having a special memory ability, NiTi was prone to separating without notice. Both torsional and flexural fractures can occur in these files. Repetitive phase transformation is produced during instrumentation by loading and unloading NiTi files.

Gavini et al. (2018) stated that after cryotreatment, nitrogen was equally distributed across the instrument's cross-section [16]. This could be explained by using two different nitrogen distribution processes with different outcomes, such as cryotherapy, depending on the composition of the alloy and the manufacturing process. Zelic et al. (2015) discovered that while NiTi compositional changes had no substantial impact on its mechanical characteristics, the thermomechanical history must be considered a key influence in the ultimate mechanical strength [4]. Because the tested files in the NiTi rotary single-file system (Procodile, Komet) were created with distinct geometric features and designs, it is challenging to assess the impact of a single factor on their cyclic fatigue resistance. The mechanical behavior of endodontic rotary files under bending and torsional circumstances has been analyzed extensively using the FE approach. The automated process makes it possible to create an FE model of the endodontic file using the previously created geometry and an accurate description of the geometry of the file based on its design parameters.

The study by Vukicevic et al. compared the cyclic fatigue resistances of single-file NiTi systems: One Shape (Micro-Mega, Besancon, France), HyFlex EDM (Coltene/Whaledent, Altstätten, Switzerland), WaveOne Gold (Dentsply Maillefer, Ballaigues, Switzerland), and Reciproc Blue (VDW, Munich, Germany) [17]. The cyclic fatigue resistance of the HyFlex EDM files was higher than that of the One Shape, Reciproc Blue, and WaveOne Gold files within the confines of the current in vitro investigation. This results from the rotary files' alloy composition, which enhances their ability to withstand cyclic fatigue [17]. Both 48 Neoniti and Reciproc NiTi rotary files (#25, 6% taper) were used in an in vitro experimental investigation by Nakamura et al. in two subgroups with and without cryogenic treatment [18]. Neoniti and Reciproc NiTi rotary files' cyclic fatigue resistance can be considerably increased by cryogenic treatment [18]. This is because changes in the crystalline nature help to reduce the cyclic fatigue of rotary files.

The study's limitations include its reliance on in vitro models, limited sample size, use of simplified tooth models, potential difficulty in generalizing findings beyond the specific experimental conditions, focus on mechanical factors, exclusive evaluation of a single-file system, and omission of clinical variables such as root canal anatomy and operator experience. Thus, great care should be taken when extrapolating the findings of this in vitro investigation. To account for geographic differences in the FEA simulation and in clinician preparation, more plastic teeth than the two employed in this study might be used in future research. However, given the constraints of this in vitro comparative investigation, it can be deduced that following numerous root canal applications, the NiTi rotary single-file system (Procodile, Komet) experienced reduced stress thanks to cryotreatment.

## Conclusions

Looking forward, future studies might consider expanding the range of plastic teeth used to better account for geographic and clinician variations. Despite the limitations of this in vitro analysis, the current findings show a significant reduction in stress within the NiTi rotary single-file system (Procodile, Komet) after multiple root canal procedures with cryotherapy. This suggests promise for improving endodontic instrument durability. While acknowledging study constraints, including its focus solely on mechanical factors and exclusion of clinical variables, the present results lay the groundwork for further investigation. Overall, the findings represent a step forward in endodontic research, offering the potential for enhanced treatment outcomes and patient care.
